# Supplementary material for: VGEA: an RNA viral assembly toolkit
Source: PeerJ. 2021 Sep 6;9:e12129. doi: 10.7717/peerj.12129 (PMC8428259; doi:10.7717/peerj.12129)
Supplement: Supplemental Information 2 [file peerj-09-12129-s002.zip › Supplementary_File_2/Performance_Comparison_Assembly_Pipelines_Table_2/CV45/vgea/quast_results/results_2021_05_13_23_04_04/genome_stats/features_frcurve_plot.pdf]

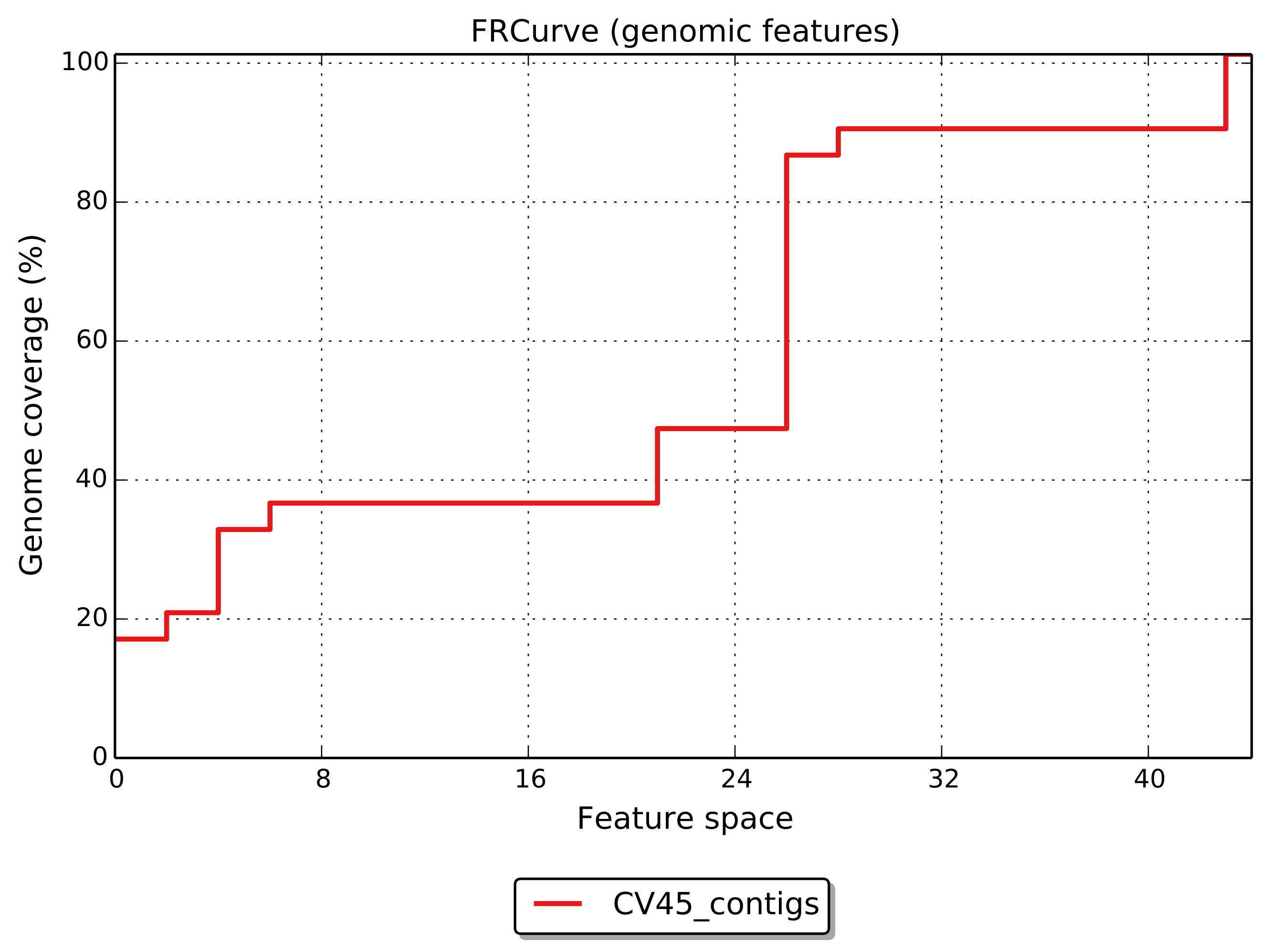
FRCurve (genomic features)
Genome coverage (%)
Feature space
0
20
40
60
80
100
0
8
16
24
32
40
CV45_contigs
